# Compressive Behavior of Circular Sawdust-Reinforced Ice-Filled Flax FRP Tubular Short Columns

**DOI:** 10.3390/ma13040957

**Published:** 2020-02-20

**Authors:** Yanlei Wang, Guipeng Chen, Baolin Wan, Baoguo Han

**Affiliations:** 1 State Key Laboratory of Coastal and Offshore Engineering, School of Civil Engineering, Dalian University of Technology, Dalian 116024, China; chenguipeng1993@163.com (G.C.); hanbaoguo@dlut.edu.cn (B.H.); 2 Department of Civil, Construction and Environmental Engineering, Marquette University, Milwaukee, WI 53201, USA; baolin.wan@marquette.edu

**Keywords:** flax fiber, FRP, sawdust, ice, tubular column, confinement

## Abstract

Sawdust-reinforced ice-filled flax fiber-reinforced polymer (FRP) tubular (SIFFT) columns are newly proposed to be used as structural components in cold areas. A SIFFT column is composed of an external flax FRP tube filled with sawdust-reinforced ice. The compressive behavior of circular SIFFT short columns was systematically investigated. Four types of short columns with circular sections, including three plain ice specimens, three sawdust-reinforced ice specimens (a mixture of 14% sawdust and 86% ice in weight), nine plain ice-filled flax FRP tubular (PIFFT) specimens and nine SIFFT specimens, were tested to assess the concept of the innovative composite columns. The test variables were the thickness of flax FRP tubes and the type of ice cores. The test results indicated that the lateral dilation and the development of cracks of the ice cores were effectively suppressed by outer flax FRP tubes, thus causing a considerable enhancement in the compressive strength. Moreover, the compressive behavior, energy-absorption capacity, and anti-melting property of sawdust-reinforced ice cores were better than those of plain ice cores confined by flax FRP tubes with the same thicknesses. The proposed equations for estimating ultimate bearing capacities of PIFFT and SIFFT short columns were shown to provide reasonable and accurate predictions.

## 1. Introduction

The low temperature in cold areas tremendously limits the use of traditional building materials, especially concrete. Water freezing causes detrimental effect on hydration reaction of the cement, resulting in severe deterioration of mechanical properties of the resulting concrete. Furthermore, the local environment will be easily polluted due to the use of concrete in construction. Therefore, there is a particular need to find a potential construction material with cost-effective and environmentally friendly advantages to replace concrete in such areas. 

In fact, the local ice has served as a cleaner construction material since ancient times in cold areas, such as the igloos built up by Inuit people. In addition, the compressive properties of ice have been investigated by many researchers (e.g., [1,2,3,4]). Ice belongs to a brittle material relatively strong under compression but very weak under tension, which is very similar to concrete [4]. However, the cylinder compressive of plain ice at −2 °C is less than 1/10 of that of normal concrete specimen with the same size [5]. Therefore, appropriate measures need to be taken to ameliorate the mechanical properties of plain ice. A variety of materials including alluvium [6], sand [7], and fiberglass net and cloth [8] were introduced into plain ice as reinforcement. The test results demonstrated that the mechanical properties of plain ice were enhanced after introducing the aforementioned materials in ice. For example, the compressive strength of the ice specimens modified by 2% volumetric content of fiberglass net was twice as large as that of the plain ice at−20 °C [8]. Moreover, sawdust is another popular reinforcing material for the modification of plain ice. Vasiliev et al. [9] studied the compressive and flexural behaviors of ice specimens reinforced by sawdust, wood chips, and wood shavings at –18 °C. The test results revealed that the compressive and bending strengths of plain ice were enhanced from 3.18 and 1.24 MPa to 12.45 and 3.74 MPa, respectively, after introducing 10.5% sawdust in weight. However, the compressive strength of the reinforced ice is still weaker than that of normal concrete, which severely restricts its further application. Furthermore, the surrounding chemicals might have a detrimental impact on the long-term mechanical properties of the reinforced ice when directly exposed to environmental attack without an outer protective jacket. Therefore, it is desired to find ways to overcome such shortcomings. 

More recently, fiber-reinforced polymer (FRP) composites have been increasingly used in civil engineering because of their high strength-to-weight ratios, good design flexibility, and excellent corrosion resistance even under harsh environment [10,11,12,13,14,15,16,17,18,19,20,21,22]. In the 1990s, Mirmiran and Shahawy [23] proposed concrete-filled FRP tubular (CFFT) columns. For a CFFT column, the pre-fabricated tube made of FRP composites acts as permanent formworks for fresh concrete without concern for possible corrosion. Furthermore, the outer FRP tube offers additional confinement to the concrete core. Many researches (e.g., [24,25,26,27,28,29,30]) have been carried out on the axial compressive behavior of CFFT columns, which demonstrated the excellent structural performance of such members. However, the majority of the reinforcing fibers in current FRP belong to synthetic fibers (e.g., carbon and glass fibers), which will consume large amounts of energy. 

In consideration of environmental protection and sustainable development, natural fibers have attracted the attention of many researchers as substitutable reinforcements for traditional synthetic fibers due to their advantages such as low cost, high specific strength, environmental friendliness and bio-degradability, ease of fabrication, and good structural rigidity [31,32,33]. Dittenber and Gangarao [34] compared over 20 common natural fibers (e.g., sisal, ramie, kenaf, jute, hemp, flax, coir, cotton, etc.) with glass fibers in terms of specific modulus, cost per weight, as well as cost per unit length (capable of resisting 100 kN load). It revealed that flax fibers possess the optimum combination of advantages among these natural fibers. More recently, flax FRP tubes were used to provide lateral confinement to concrete columns (e.g., [35,36,37]). Yan and Chouw [35] investigated the axial compressive performance of the coir fiber-reinforced concrete-filled flax FRP tubes, which revealed that the compressive strength and the ductility of the coir fiber-reinforced concrete cores were substantially improved by the additional confinement from flax FRP tubes. Xia et al. [37] studied the behavior of self-compacting concrete-filled flax FRP tubular columns under static and cycle compression, which confirmed that the compressive strength and the ultimate axial strain of filament-wound flax FRP-confined concrete were increased with the increase of tube thickness. In view of the similarities between ice and concrete, it might be a good way to study the compressive responses of the flax FRP-confined ice based on the well understanding of the behavior of flax FRP-confined concrete.

Based on previous researches on sawdust-reinforced ice [9] and concrete-filled flax FRP tubes (e.g., [35,36,37]), sawdust-reinforced ice-filled flax FRP tubular (SIFFT) columns are innovatively proposed in this paper to dramatically improve the compressive properties of plain ice. They are composed of flax FRP tubes filled with sawdust-reinforced ice. The compressive strength of inner ice is expected to be greatly improved by the flax FRP confinement. In addition, the melting of inner ice might be delayed due to the inclusion of sawdust. Furthermore, the isolation of flax FRP tube, with a low thermal conductivity of around 0.04 W/(m·K) for flax fibers [38], might also contribute to the improvement of thermal insulation property. Additionally, the flax FRP tubes can be filled with sea ice without any corrosion concern, which is a significant advantage over traditional steel tubes. Finally, the proposed SIFFT column is a novel structural member with less environmental pollution: (a) ice is a clean material, readily available in cold areas, which becomes water after melting without pollution [39]; (b) natural fibers (i.e., flax fibers and wood waste sawdust) used in this study are biodegradable materials. The novel SIFFT columns are expected to be potentially used in the piers of a bridge built in remote cold regions. Moreover, the outer steel columns of the structures of the existing scientific research station in polar areas might be replaced by the proposed SIFFT columns with better durability.

Experimental studies were conducted to assess the axial compressive behavior of the newly proposed SIFFT column and to verify the possibility of this column to be used as a compression member in cold regions. Effects of the thickness of flax FRP tube and the inclusion of sawdust in ice were discussed. Equations were proposed to predict the ultimate bearing capacities of circular plain ice-filled flax FRP tubular (PIFFT) short columns and circular sawdust-reinforced ice-filled flax FRP tubular (SIFFT) short columns.

## 2. Experimental Program 

### 2.1. Specimens

A total of 18 circular short composite columns, including nine plain ice-filled flax FRP tubular (PIFFT) specimens and nine sawdust-reinforced ice-filled flax FRP tubular (SIFFT) specimens, were tested subjected to axial compression. In addition, three unconfined plain ice specimens and three unconfined sawdust-reinforced ice specimens were prepared as control specimens. Each series included three nominally identical specimens. All test specimens had a height of 300 mm and a nominal diameter of 150 mm, measured at the surface of ice cores. The test variables included the number of FRP layers in flax FRP tubes and the type of ice cores. It has been well established that the compressive property of ice will be improved after the inclusion of sawdust in ice due to the fiber bridging effect. However, it does not mean that more sawdust will always contribute to better compressive property of the sawdust-reinforced ice. In fact, the workability of the water–sawdust mixture will decrease when the volume fraction of sawdust exceeds a certain level, leading to more initial defects in the prepared sawdust-reinforced ice. Therefore, a decrease in the compressive property of the sawdust-reinforced ice might occur corresponding to such a large reinforcing amount. Based on the results of trial tests on sawdust-reinforced ice specimens with different volume fractions of sawdust (i.e., 14.3%, 19.8%, 26.6%, and 32.8%, respectively), the sawdust-reinforced ice with a volume fraction of 26.6% sawdust was confirmed to have the best compressive properties. Based on conversion, the optimal weight ratio of the sawdust and water was 14:86 corresponding to this volume fraction. Therefore, the sawdust-reinforced ice used in this study was a mixture of 14% pine sawdust with a dry density of 0.45 g/mm^3^ and 86% water in weight. Table 1 shows the details of all test specimens. 

The naming rule of test specimens was described as follows. (1) It began with PI, SI, IF, and SF to indicate different specimen series (i.e., plain ice column, sawdust-reinforced ice column, PIFFT column, and SIFFT column, respectively). (2) The Arabic numeral denoted the number of plies of fiber fabric in flax FRP tubes. (3) The Roman numeral was introduced to distinguish three nominally identical specimens. Taking specimen SF2-I as an example, it represented an SIFFT specimen with two FRP layers in flax FRP tube and it was the first specimen within the same series. 

### 2.2. Material Properties

Unidirectional flax fiber sheet with a surface density of 210 g/m^2^ was supplied by Nanjing Hitech Composites Co., Ltd., China. The nominal thickness of the sheet was 0.135 mm. The matching epoxy resin was obtained from Shanghai Sanyou Resin Co., Ltd., China. Considering that the proposed composite column would serve in cold areas, this type of epoxy resin was selected in this study due to the fact that its mechanical properties are not sensitive to the low temperature. However, low temperature will affect the viscosity and curing properties of the selected epoxy resin. So, the flax FRP tubes used in the paper were fabricated and cured at room temperature. The epoxy L-500 AS (main agent) was assisted by the hardener L-500 BS with a mix ratio of 2:1. According to ASTM D3039/D3039M-17 [40], flat coupon tensile tests were conducted to determine the mechanical properties of flax FRP. The test results showed that the tensile stress vs. strain featured approximately linear shape, which was similar to conventional FRP with synthetic fibers. The average elastic modulus $E_{f}$, tensile strength $f_{u}$, and ultimate tensile strain $\varepsilon_{f}$ of the flax FRP used in this study were 72.3 GPa, 784 MPa, and 1.34%, respectively. The average and standard deviation of elastic modulus $E_{f}$, tensile strength $f_{u}$, and ultimate tensile strain $\varepsilon_{f}$ of the flax FRP used in this study were 72.3 ± 3.43 GPa, 784 ± 54.0 MPa, and 1.34% ± 0.16%, respectively. It was noticed that the material properties including the elastic modulus and the tensile strength of the flax FRP in this paper are much larger than the test results reported by some researchers [e.g., 35–37], which can be explained as follows. It is difficult to precisely control the actual thickness of FRP specimens when they are prepared by hand layup process. In order to better evaluate the tensile properties of FRP fabricated by hand layup process, the tensile stress of FRP is generally calculated through the tensile load divided by the nominal thickness (ignoring the thickness of resin matrix) of the fiber fabric instead of the actual thickness (considering the thickness of resin matrix) of FRP laminate [41], thus trying to eliminate the discreteness caused by the variation of resin thickness during hand layup process. This calculation method is also adopted in this paper. In this way, both the tensile strength and the deduced elastic modulus reported in this study are overestimated compared to their true values. One may argue that the nominal modulus is larger than its true value. However, such treatment is widely accepted especially when the FRP tube prepared via wet layup process is used as a confining jacket (e.g., FRP-confined concrete members) because the nominal thickness and the nominal elastic modulus of FRP make up a couple and always appear simultaneously in the equation of determining the confining stress of the FRP tubes. Such treatment will effectively eliminate the difference and is more familiar to the engineers during the design of a certain member confined by FRP.

In addition, in order to evaluate the bearing contribution of outer flax FRP tubes in composite columns, two nominally identical flax FRP rings with three different thicknesses, with a height of 50 mm and an inner diameter of 150 mm, were prepared and axially loaded until failure following Chinese standard GB/T 5350-2005 [42]. The naming rule of flax FRP ring specimens was described as follows. It began with FR representing FRP rings, followed by a number indicating the number of FRP layers in flax FRP rings. The last Roman numeral was used for differentiating two nominally identical specimens. The test results of the flax FRP rings were presented in the next section.

### 2.3. Preparation of Specimens

**Figure 1:**
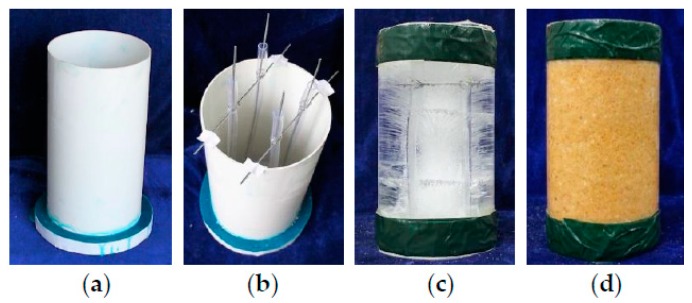
Preparation of plain ice and sawdust-reinforced ice specimens: (**a**) PVC formwork; (**b**) placement of hoses; (**c**) plain ice specimen; and (**d**) sawdust-reinforced ice specimen.

The main preparation processes of plain ice and sawdust-reinforced ice specimens included the following steps: (1) A PVC tube with an inner diameter of 150 mm and a height of 300 mm was capped with a wooden plate by epoxy resin, which was used as the formwork for preparing ice specimens (Figure 1a). (2) Four hollow PVC hoses with sealed bottoms were symmetrically placed in the formwork (Figure 1b). The volume expansion during ice formation would squeeze the voids of the hollow hoses. Therefore, the radical pressure during water freezing process would be significantly released, leading to a great decrease in the number of cracks in ice [5,43]. (3) For sawdust-reinforced ice specimens, the sawdust was uniformly mixed with the water at the weight ratio of 14 (the sawdust) to 86 (the water) via mechanical stirrer. Considering the high water absorption of sawdust, the mixture should stay for at least three hours at ambient temperature to ensure that the sawdust was fully saturated in water. (4) Water and the mixture of sawdust and water were poured into the formworks for plain ice and sawdust-reinforced ice specimens, respectively. For the sawdust-reinforced ice specimen, the mixture of sawdust and water should be compacted layer by layer (five layers in total) to avoid bubbles. (5) After the casting was finished, the formworks were placed in the refrigerator (AUCMA Inc., Qingdao, China) at −15 °C for 48 h. (6) Finally, five plies of 45-mm-wide duct tapes were used to strengthen the ends of plain ice column (Figure 1c) and sawdust-reinforced ice column (Figure 1d) to avoid any premature failure at such locations due to stress concentration during the test. As shown in Figure 1d, the sawdust was approximately evenly distributed in the sawdust-reinforced ice specimen corresponding to the selected volume fraction (i.e., 26.6%).

**Figure 2:**
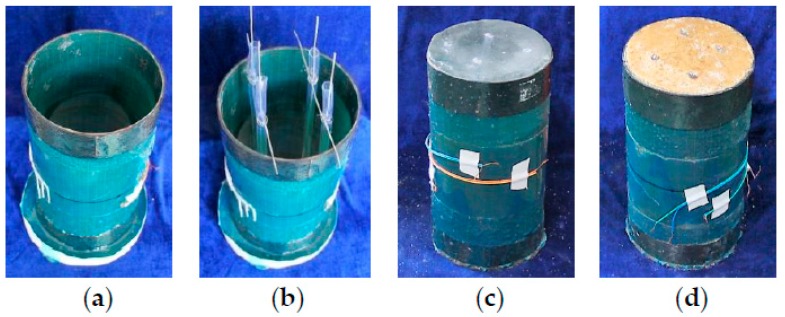
Preparation of plain ice-filled flax fiber-reinforced polymer tubular (PIFFT) and sawdust reinforced ice-filled flax fiber-reinforced polymer tubular (SIFFT) specimens: (**a**) flax fiber-reinforced polymer (FRP) tube; (**b**) placement of hoses; (**c**) PIFFT specimen; and (**d**) SIFFT specimen.

The preparation of PIFFT and SIFFT specimens was similar to that of aforementioned unconfined ice specimens. Firstly, the flax FRP tube was prepared via wet layup process by wrapping resin-impregnated flax fiber fabric around a cylindrical steel mold with fibers oriented in the hoop direction, with an overlapping length of 150 mm. Each end of flax FRP tube was strengthened by a 45-mm-wide carbon FRP strip to avoid any premature failure at the ends. In order to constitute a formwork, one end of the prepared flax FRP tube was capped with a circular wooden plate (Figure 2a). The following procedures, including the installation of hollow hoses (Figure 2b), the casting of water or the mixture of sawdust and water into flax FRP tubes, and the freezing in refrigerators were the same as those of unconfined plain ice and sawdust-reinforced ice columns.

### 2.4. Test Setup and Instrumentation

**Figure 3:**
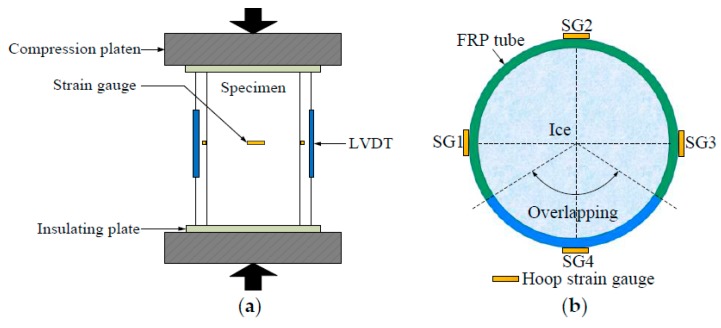
Test setup and instrumentation: (**a**) Test setup and linear variable displacement transducers (LVDTs); and (**b**) layout of strain gauges.

All axial compression tests were conducted in winter. Prior to the compression test, the temperature of all specimens should be stabilized at −3 °C, which is equal to the temperature inside the laboratory, to avoid the heat exchange between the specimens and surrounding environment during the axial compression loading. It should be admitted that the difference between the specimen preparation temperature (i.e., −15 °C) and specimen compression temperature (i.e., −3 °C) might cause some internal damage of the ice, which was ignored in this study. A more accurate experimental program remained to be conducted in the authors’ future study to take this influence into consideration. All specimens were axially loaded under displacement control at a loading rate of 1.5 mm/min. As shown in Figure 3a, two insulating plates were placed between the testing machine and specimen’s each end to block the heat transfer between them [39]. Two linear variable displacement transducers (LVDTs, Liyang Instrument and Meter Inc., Liyang, China) were installed to monitor the overall axial shortening of the specimen (Figure 3a). In addition, for each PIFFT or SIFFT specimen, four strain gauges (Zhejiang Huangyan Testing Instrument Inc., Taizhou, China) with a gauge length of 20 mm were mounted on the surface of the outer flax FRP tube at the mid-height region to monitor the hoop strains. One (i.e., SG4) was placed within the overlapping zone and the remaining three (i.e., SG1, SG2, and SG3) were symmetrically distributed at 90° outside the overlapping zone (Figure 3b).

### 2.5. Melting Tests

**Figure 4:**
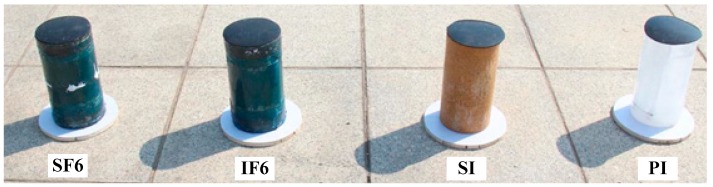
Melting specimens.

In addition to axial compression tests, melting tests were also designed in this study to evaluate the melting rate of different types of specimens. As shown in Figure 4, four representative specimens, including plain ice short column (PI), sawdust-reinforced ice short column (SI), PIFFT short column (IF6), and SIFFT short column (SF6), were placed on the roof of the laboratory to conduct the melting tests. The specimen naming rule was identical to that proposed for the specimens in the compression tests. For PIFFT and SIFFT specimens, considering that the heat insulation effect might be better when a thicker FRP tube was used, flax FRP tubes with six FRP layers were used in the melting tests. A circular wooden plate was placed on the bottom of each specimen to avoid direct contact between the specimen and the ground. Moreover, a rubber cap with a diameter of 160 mm was set on the top of each specimen to avoid direct sunlight based on the consideration that the top surface of the ice column in an actual structure should not be directly exposed to the surrounding environment. The melting tests were carried out from March 22, 2018 at 10:00 to March 25, 2018 at 13:00. The outdoor temperature and the weight of each specimen were simultaneously recorded twice per hour in the first 12 h and then once per hour in the following 63 h.

## 3. Results and Discussions 

### 3.1. General Observations

**Figure 5:**
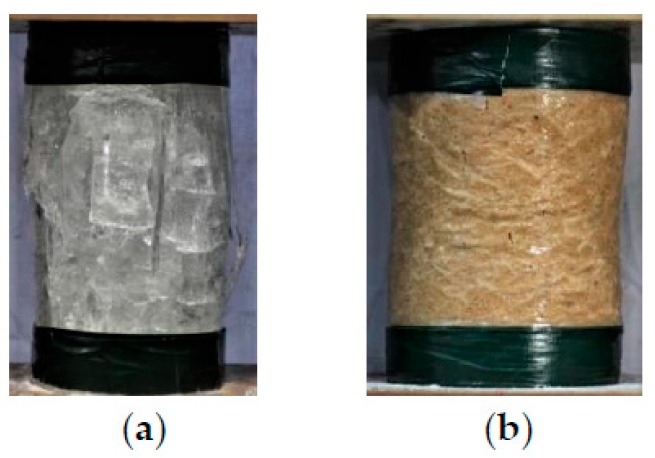
Typical failure modes of (**a**) plain ice specimen and (**b**) sawdust-reinforced ice specimen.

Many longitudinal cracks were developed through the whole height of the plain ice specimen during compression, which was followed by significant dilation near the mid-height region at failure (Figure 5a). By contrast, the number of cracks in the sawdust-reinforced ice specimen after the compression tests was dramatically decreased (Figure 5b). This might be interpreted by sawdust fiber bridging effect, which played a significant role on reducing and holding cracks in ice. In addition, pronounced dilation occurred near the ends of sawdust-reinforced ice specimen, while the lateral deformation at mid-height region was relatively small, which was probably attributed to the excellent plastic deformability of reinforced ice after the modification of sawdust.

**Figure 6:**
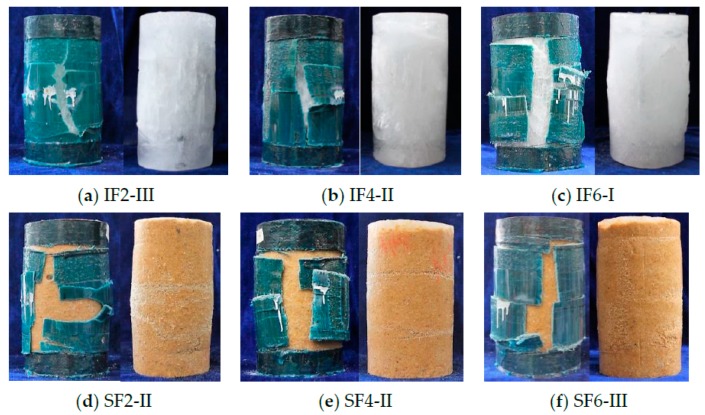
(**a**–**f**) Typical failure modes of PIFFT and SIFFT specimens (IF and SF representing PIFFT and SIFFT, respectively; Arabic numeral representing the number of flax FRP layers; and Roman numeral differentiating three nominally identical specimens).

The typical failure modes of PIFFT and SIFFT specimens after the compression tests are shown in Figure 6. Both PIFFT and SIFFT specimens failed by the sudden rupture of flax FRP tubes outside the additional confinement zone at two ends accompanied by a loud popping noise, which was very similar to the failure mode of concrete-filled flax FRP tubular (CFFT) short columns under axial compression reported by Xia et al. [44]. The flax FRP tubes were carefully removed after finishing the tests. The lateral dilation of confined plain ice at mid-height was observed to be much smaller than that of unconfined plain ice because of the effective confinement of the outer FRP tube. Some minor longitudinal cracks developed in the plain ice cores corresponding to the rupture zone of the outer flax FRP tube. By contrast, few cracks were observed in sawdust-reinforced ice core. Furthermore, the dilation of confined sawdust-reinforced ice near mid-height region was more significant than that of each end due to the additional confinement from the carbon FRP strip, which was tremendously different from the failure mode of unconfined sawdust-reinforced ice specimen (Figure 5b).

### 3.2. Axial Load vs. Strain Response

**Figure 7:**
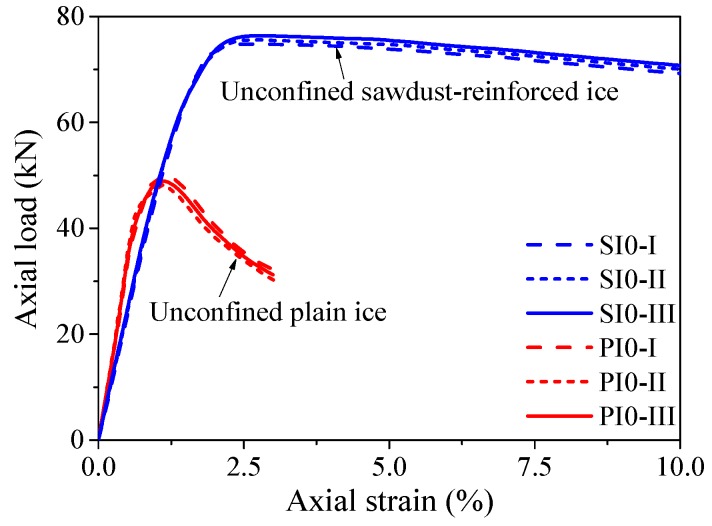
Axial load vs. strain responses of the plain ice and sawdust reinforced ice columns.

Figure 7 shows the axial load vs. axial strain responses of the plain ice and sawdust-reinforced ice specimens. Unless otherwise specified, the axial strain in this study was obtained based on the total shortening of the test specimen. As shown in Figure 7, the initial compressive stiffness of the plain ice specimens was slightly higher than that of the sawdust-reinforced ice specimens. The average elastic moduli of the plain ice and sawdust-reinforced ice were 0.36 and 0.28 GPa, respectively. The average bearing capacity and the corresponding axial strain of the plain ice specimens were 48.98 kN and 1.13%, respectively, while 75.61 kN and 2.41% for the sawdust-reinforced ice specimens. As for the descending branch, the axial load of the sawdust-reinforced ice specimens decreased much more slowly than that of the plain ice specimens, which confirmed that the ductility of ice was considerably improved due to the introduction of sawdust. Overall, the sawdust-reinforced ice column was observed to have higher bearing capacity and better deformability compared with the plain ice column when tested under axial compression. The compression tests were continued until the resistance of the plain ice specimens dropped to around 65% of the peak load, while the compression tests on the sawdust-reinforced specimens were terminated manually when the axial shortening reached around 30 mm due to their excellent deformability.

In a concrete-filled FRP tubular (CFFT) column, the axial bearing contribution of the outer FRP tube to the total load carried by the composite column would be very limited if the fibers are mainly oriented in the hoop direction. In fact, the compressive strength of an FRP tube in the orthogonal direction to the reinforcing fibers is mainly dependent on its resin matrix [45]. Therefore, the axial contribution of such FRP tube to a CFFT column is usually omitted during the calculation process. However, given that the elastic moduli of unconfined plain ice (0.36 GPa) and sawdust-reinforced ice (0.28 GPa) tested in this study were much smaller than that of the normal concrete (around 30 GPa), the compressive bearing contribution of outer flax FRP tubes in PIFFT and SIFFT columns should be taken into consideration.

**Figure 8:**
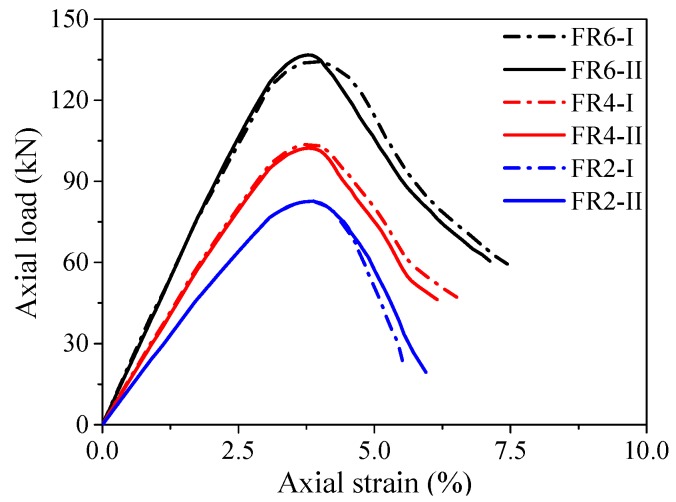
Axial load vs. strain responses of flax FRP rings.

It was observed that the local buckling of the outer flax FRP tube in PIFFT and SIFFT specimens was effectively suppressed by the corresponding plain ice and sawdust-reinforced ice cores before rupture. Similarly, the local buckling of the axially loaded flax FRP rings was also not observed before reaching their peak loads. In the present study, it was assumed that the compressive behavior of the outer flax FRP tube in PIFFT and SIFFT columns can be reflected by the axial load vs. axial strain curves of the flax FRP ring specimens (Figure 8). As shown in Figure 8, the initial compressive stiffness and the bearing capacity of the flax FRP rings increased with the wall thickness. The average bearing capacities of the flax FRP rings with two, four, and six FRP layers were 82.66, 103.01, and 135.56 kN, respectively. The axial strains at peak loads of the flax FRP rings with three different numbers of FRP layers, with an average value of 3.84%, were close to each other. It was obvious that the peak loads of the flax FRP rings were larger than those of the unconfined plain (i.e., 48.98 kN) and sawdust-reinforced ice (i.e., 75.61 kN) specimens, which confirmed the necessity of considering the compressive contribution of outer flax FRP tubes to composite columns in this study. 

**Figure 9:**
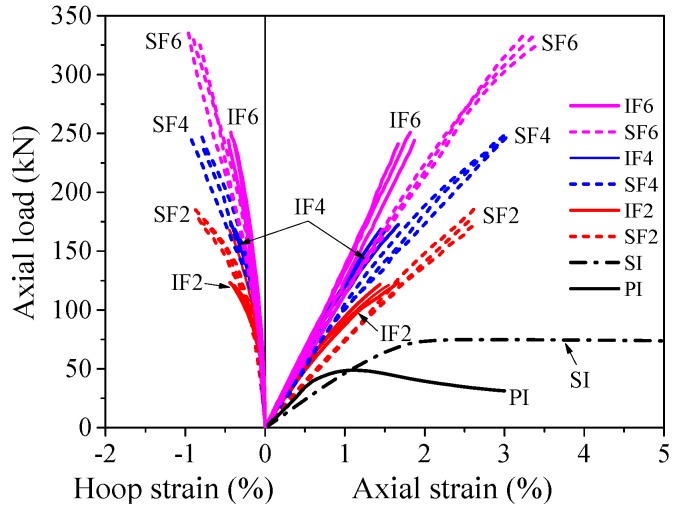
Axial load vs. strain responses of PIFFT and SIFFT specimens.

Figure 9 shows the axial load vs. strain responses of PIFFT and SIFFT columns. The hoop strain was the average value of the readings of three hoop strain gauges (i.e., SG1, SG2, and SG3) outside the overlapping zone (Figure 3b). The ultimate condition of the composite column was characterized by the hoop rupture of outer flax FRP tubes in PIFFT and SIFFT columns. The axial load vs. axial strain responses of composite columns terminated at the peak point when the absolute value of hoop strain reached its maximum value (i.e., hoop rupture strain). The peak loads of PIFFT and SIFFT specimens were obviously greater than those of the corresponding unconfined plain ice and sawdust-reinforced ice column, respectively. As shown in Table 1 and Figure 9, the peak loads of PIFFT and SIFFT specimens increased with the increasing thickness of outer flax FRP tubes. In addition, the peak load, hoop rupture strain, and ultimate axial strain of SIFFT column were obviously larger than that of PIFFT column with the same tube thickness. It should be noted that the deformability of the unconfined sawdust-reinforced ice specimen (within SI series) was so excellent that its axial strain was just plotted from 0% to 5% in Figure 9. 

### 3.3. Axial Stress vs. Strain Behavior of Confined Ice

**Figure 10:**
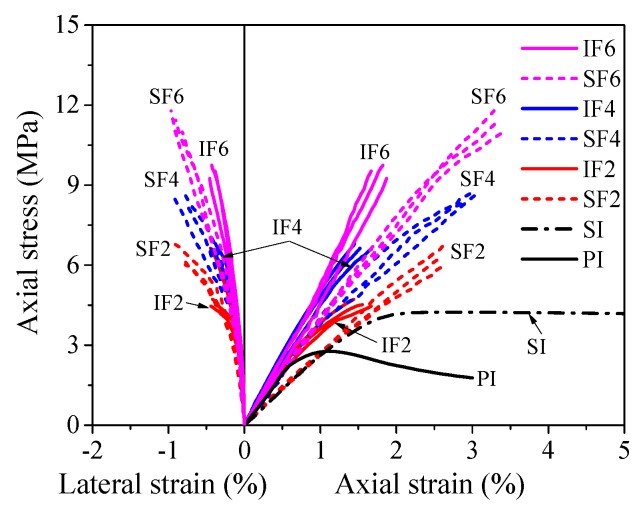
Axial stress vs. strain responses of the confined plain ice and sawdust-reinforced ice.

The axial load was simultaneously shared by the outer flax FRP tube and the plain or sawdust-reinforced ice core when a PIFFT or SIFFT column was tested under axial compression. Based on the static equilibrium as well as the axial deformation compatibility between outer tube and inner ice, the load carried by the ice core was believed to be equal to the difference between the load resisted by the composite column and the load carried by the flax FRP tube at the same axial strain, and the latter was obtained through the compression tests on flax FRP rings (Figure 8). It is worth mentioning that the hollow flax FRP ring specimens and the outer flax FRP tubes in composite columns had different stress states, because the latter also suffered hoop tension apart from axial compression. However, this distinction was ignored for simplification in this study. Based on the simplified assumption, the axial stress vs. axial strain responses of the flax FRP-confined plain ice and sawdust-reinforced ice were obtained, as shown in Figure 10.

In terms of hoop strain, in an FRP-confined concrete column, the FRP jacket and inner concrete were believed to have compatible deformation in the circumferential direction. In this study, the hoop strain of the flax FRP tube in PIFFT and SIFFT columns was also employed to assess the lateral strain of the confined plain ice and sawdust-reinforced ice due to the fact that the inner ice was well confined by the outer flax FRP tube until rupture. The axial stress vs. lateral strain responses of the plain ice cores and sawdust-reinforced ice cores are also exhibited in Figure 10 to demonstrate their lateral dilation during the loading process. 

The main test results of the plain ice cores and sawdust-reinforced ice cores in PIFFT and SIFFT specimens are summarized in Table 2, including the axial compressive strength $f_{ic}^{\prime }$, ultimate axial strain $\varepsilon_{iu}$, and the hoop rupture strain $\varepsilon_{h,rup}$ of outer flax FRP tubes. The strength and strain enhancement ratios (i.e., $f_{ic}^{\prime }/f_{io}^{\prime }$ and $\varepsilon_{iu}/\varepsilon_{io}$), together with the ratios between hoop rupture strains of flax FRP tubes and material ultimate tensile strains of flat coupons (i.e., $\varepsilon_{h,\mathrm{rup}}$/$\varepsilon_{f}$), are also shown in Table 2. It can be seen that the strength enhancement ratio of confined sawdust-reinforced ice was smaller than that of the confined plain ice corresponding to the same number of flax FRP layers. Moreover, the strain enhancement ratio was observed to have a similar trend. Additionally, the average hoop rupture strain of flax FRP tubes in SIFFT specimens was around twice as large as that of flax FRP tubes in PIFFT specimens.

As shown in Table 2, the compressive strengths of the confined plain ice and sawdust-reinforced ice were obviously greater than those of the corresponding unconfined plain ice and sawdust-reinforced ice, and they increased approximately linearly with the number of flax FRP layers in this study, which was also observed in self-luminous glass FRP-confined ice [39]. In addition, the compressive strengths and peak axial strains of the unconfined and confined sawdust-reinforced ice were larger than those of the corresponding unconfined and confined plain ice with the same thickness of flax FRP tube, which indicated the compressive properties of ice would be effectively enhanced after the introduction of sawdust. It was noticed that the number of FRP layers in flax FRP tubes had only a marginal effect on the peak strains of the confined plain ice, while a slightly linear increase was observed for confined sawdust-reinforced ice with the increasing flax FRP layers. Overall, the axial compressive properties including compressive strength and corresponding peak axial strain of the sawdust-reinforced ice were better than those of the plain ice regardless of the existence of flax FRP tubes.

### 3.4. Lateral Dilation Behavior of Confined Ice

**Figure 11:**
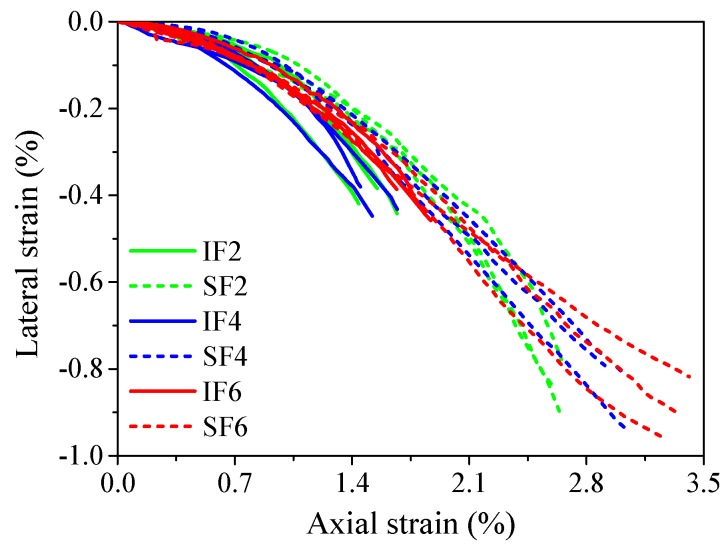
Lateral strain vs. axial strain responses of confined ice.

It is generally accepted that the lateral dilation (hoop strain) of confined concrete is a function of the axial strain. Meanwhile, the dilation of the concrete core is restricted by the confining pressure provided by steel stirrups and/or FRP jackets. Therefore, the confining pressure is dependent on the axial strain and some analytical models have been established based on this understanding [46,47,48]. Similarly, the development of the confining pressure provided the flax FRP tube in this study is also expected to be a function of the axial strain of the confined ice. Considering that the flax FRP is a linear elastic material, as a simplification, the relationship between the lateral strain (instead of confining pressure from the flax FRP tube) and the axial strain of the plain ice cores and sawdust-reinforced ice cores are presented in Figure 11. It was observed that the lateral strain vs. axial strain responses of all ice cores were close to each other when their axial strains were within 0.5%. After that, the absolute values of the lateral strains of the plain ice cores were greater than those of sawdust-reinforced ice cores at a given axial strain, especially for plain ice cores confined by two and four FRP layers in flax FRP tubes. This observation indicated that the lateral dilation of the confined sawdust-reinforced ice was more effectively suppressed due to the fiber bridging effect after the introduction of sawdust in ice.

### 3.5. Hoop Strain Distribution of FRP Tube

**Figure 12:**
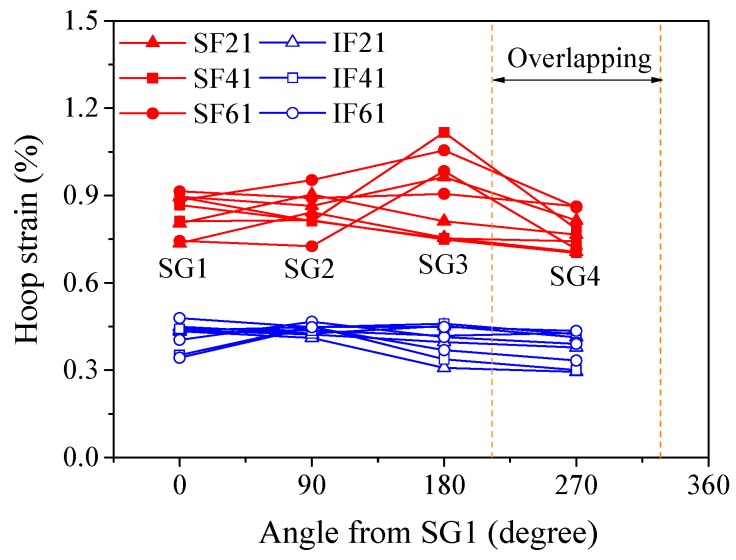
Distribution of hoop strains of FRP tubes in PIFFT and SIFFT specimens at peak loads.

The uniformity of the lateral dilation of flax FRP-confined might be reflected by the hoop strain distribution of outer flax FRP tube around the circumference. Figure 12 shows the distribution of the hoop strains of flax FRP tubes in PIFFT and SIFFT specimens corresponding to the peak loads. The hoop strains were monitored by the four hoop strain gauges around the circumference of the outer flax FRP tube (Figure 3b). It was observed that the hoop strain distribution of flax FRP tubes in SIFFT specimens was more non-uniform compared with that in PIFFT columns. Similarly, the hoop strain distribution of FRP jacket in FRP-confined concrete columns tested under axial compression was also observed to be non-uniform [41]. It might be attributed to the fact that both the sawdust-reinforced ice and the concrete are a mixture of at least two constituent materials, and there is an unavoidable regional heterogeneity in the body of the specimen even though it is carefully prepared. By contrast, the plain ice was prepared using one component material (i.e., water), thus causing a much more uniform distribution of the hoop strains of flax FRP tubes in PIFFT columns in this study. In addition, the hoop strains within the overlapping zone of flax FRP tubes in PIFFT and SIFFT specimens were observed to be slightly smaller than those outside the overlapping zone, which was similar to the observations in axially loaded FRP-confined concrete columns [41].

**Figure 13:**
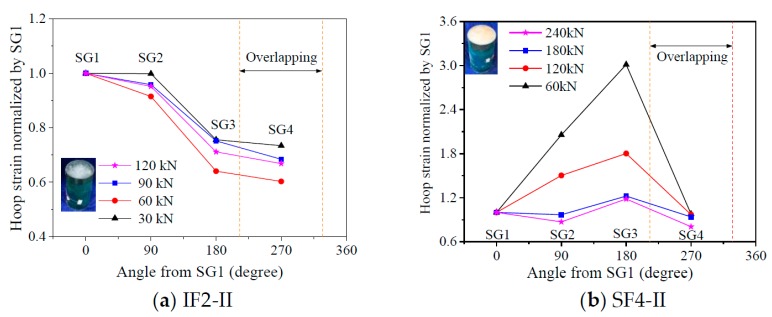
Distribution of normalized hoop strains of FRP tubes in PIFFT and SIFFT specimens at different loads.

It was also of great significance to investigate the hoop strain distribution of outer flax FRP tubes in the composite columns at different loading period. In this study, specimens IF2-II and SF4-II were selected as representatives to evaluate the distribution of the hoop strains of the flax FRP tubes in PIFFT and SIFFT specimens at different loading level, respectively. In order to clearly present the variation of hoop strains obtained even at the smallest axial load, all hoop strains were normalized by the readings of SG1 at each level of loading. As shown in Figure 13a, the normalized hoop strain distribution curves of flax FRP tubes in PIFFT specimens generally had a similar trend regardless of the variation of axial load. By contrast, the normalized hoop strain distribution in SIFFT specimens (Figure 13b) was observed to become more uniform with the increase of axial load. The phenomenon might be explained by the fact that the confined sawdust–ice mixture with more initial defects than plain ice becomes denser with the increase of axial load and lateral confinement. As a result, a more evenly distributed hoop strains were observed in the more homogeneous sawdust-reinforced ice with the increasing loading level. Moreover, the hoop strains within the overlapping zone of FRP tubes in PIFFT and SIFFT columns were smaller than those outside the overlapping zone at different loading level.

### 3.6. Energy-Absorption Capacity

As we all know, a good structural member is required to have high bearing capacity, good deformability, and large energy-absorption capacity. In this study, the flax FRP composites were almost elastic until failure without yielding characteristic and the axial load vs. strain responses of PIFFT and SIFFT columns were approximately linear before reaching the ultimate state. Therefore, it was unsuitable to use peak load or ultimate axial strain alone to evaluate the energy-absorption capacity of the composite columns. In order to solve this problem, one parameter termed energy index was initially proposed by Yan and Chouw [36] for flax FRP-confined concrete. In this paper, it was defined as the ratio of the fracture energy of the confined plain ice or sawdust-reinforced ice divided by the fracture energy of the unconfined plain ice. The fracture energy was the area under axial stress (from zero to peak value) vs. strain curve of confined ice. It was observed that the area of the confined sawdust-reinforced ice in SIFFT specimen was obviously larger than that of the confined plain ice in the corresponding PIFFT specimen with the same tube thickness.

**Figure 14:**
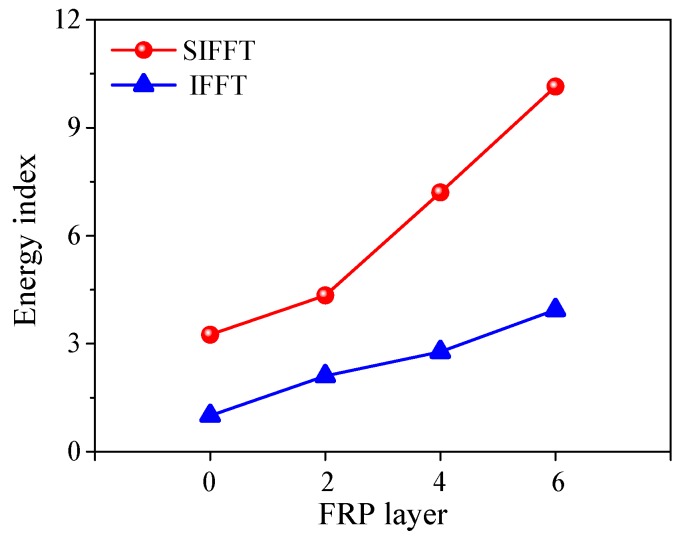
Energy index of confined plain ice and sawdust-reinforced ice.

The average energy indexes of confined plain ice and sawdust-reinforced ice are shown in Figure 14. According to the definition, the value of the energy index for the unconfined plain ice specimens was equal to one. The energy index of the unconfined sawdust-reinforced ice specimens was 3.25 times as large as that of the unconfined plain ice specimens. The energy indexes of the confined body in PIFFT and SIFFT specimens were roughly linearly increased with the increase of flax FRP layers. In addition, the energy index of the confined sawdust-reinforced ice in the SIFFT specimen was larger than that of the confined plain ice in the corresponding PIFFT specimen with the same tube thickness. The energy indexes of the confined plain ice in PIFFT specimens with two, four, and six plies of FRP layers were 2.10, 2.77, and 3.94, respectively, while 4.34, 7.20, and 10.14 for the confined sawdust-reinforced ice with the same tube thickness, respectively. In summary, it confirmed that the energy-absorption capacity of confined ice would be considerably improved if sawdust was introduced into ice or a thicker flax FRP tube was used.

### 3.7. Melting Rate

**Figure 15:**
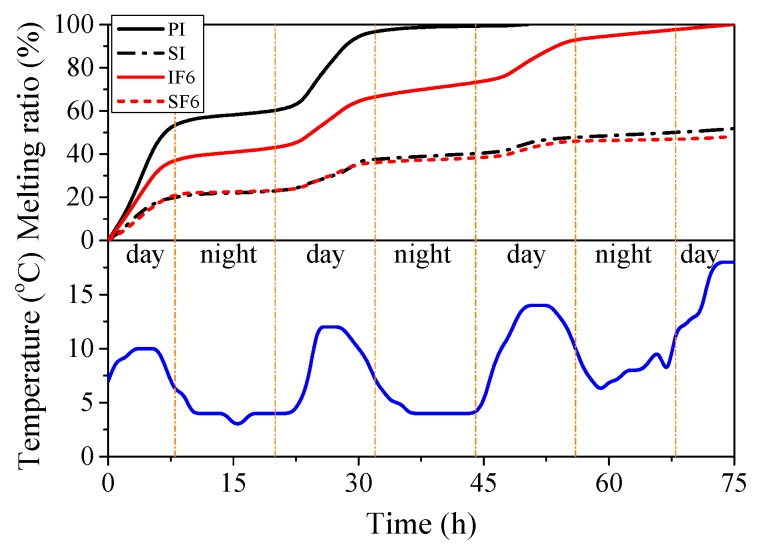
Melting rate of representative specimens and variation of temperature with increasing time.

Figure 15 shows the melting ratio of four representative specimens (Figure 4) and the corresponding outdoor temperature with increasing time periods. The melting ratio was defined as the weight reduction of each specimen divided by its original weight of its involved ice. The weights of the non-melting bodies (i.e., PVC hoses, sawdust and flax FRP tubes) were deducted during the calculation. As shown in Figure 15, during the daytime, the melting rates of the four specimens were relatively fast due to the solar radiation and increase of outdoor temperature, especially at the initial stage of the melting tests. At night, the melting rates of the specimens were much slower because of the absence of sunlight and lower temperature (around 4 °C). Moreover, the melting rate of the specimen PI was faster than that of the specimens SI and IF6, which confirmed that the melting of plain ice was effectively delayed by the inclusion of sawdust as well as the insulation of outer flax FRP tube.

**Figure 16:**
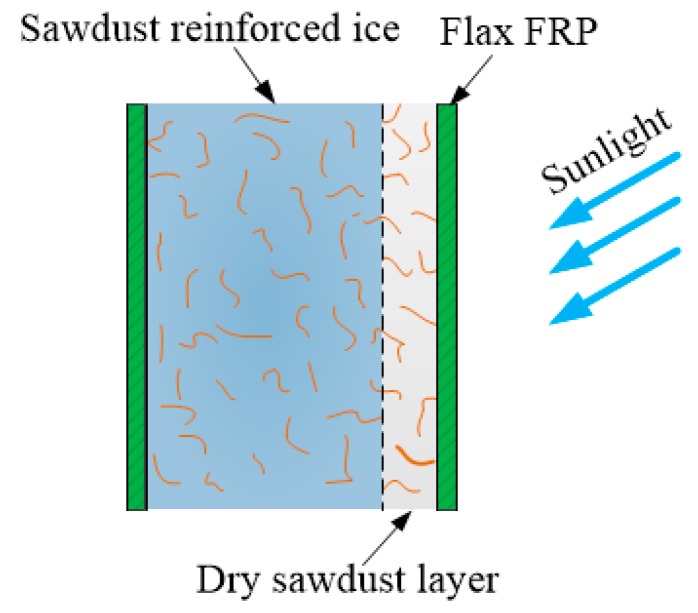
Melting mechanism of sawdust-reinforced ice in SIFFT specimen.

In addition, the melting rates of specimens SI and SF6 were close to each other, which indicated that flax FRP tube has little influence on the melting rate of sawdust-reinforced ice specimen. This phenomenon might be explained by the melting mechanism of sawdust-reinforced ice in SIFFT column, as shown in Figure 16. The unconfined or confined sawdust-reinforced ice was surrounded by a layer of dry wood fiber with a very low thermal conductivity after the initial melting, which insulated the inner frozen sawdust-ice mixture from solar radiation. Sawdust-reinforced ice specimen itself had so excellent melting-resistant properties that the insulting effect of the outer flax FRP tube in SIFFT specimen was not dominant. The specimen PI was totally melted at 50.26 h while 74.93 h for the specimen IF6. The melting rates of the specimens SI and SF6 were so slow that their monitoring was stopped at 75 h, with the corresponding final melting ratios of 51.7% and 48.3%, respectively. Finally, it should be noted that the melting test is a relatively rough exploratory test. In addition to the temperature, many other factors such as the heat flow are not taken into consideration in this paper due to the limitation of experimental conditions, which will be improved by the authors in future study.

## 4. Ultimate Bearing Capacity of Short Columns

**Figure 17:**
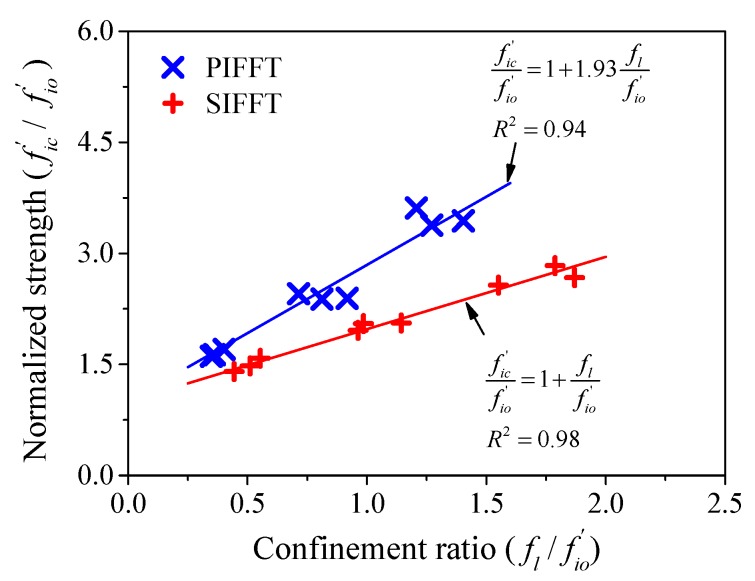
Regression of the compressive strength of confined ice.

**Figure 18:**
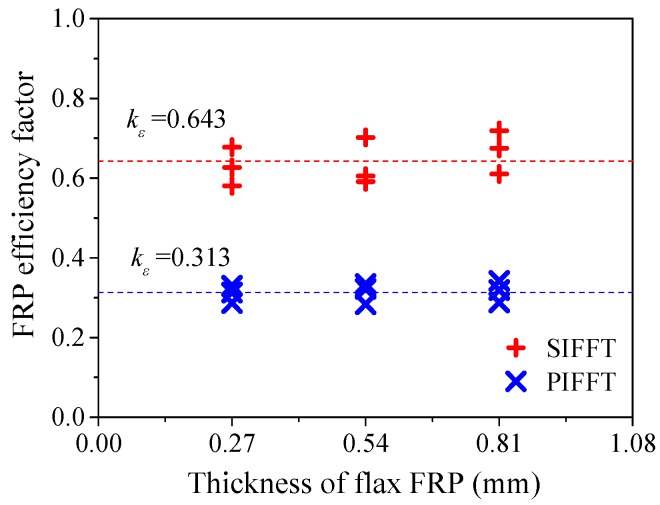
Strain efficiency factor in PIFFT and SIFFT specimens.

Taking the bearing contribution of outer flax FRP tubes into consideration, the ultimate bearing capacity $N_{u}$ of the proposed PIFFT and SIFFT short column with circular sections can be calculated as following:

(1) $$N_{u}=\beta f_{f}A_{f}+f_{ic}^{\prime }A_{i}$$

in which $A_{f}$ and $A_{i}$ = actual cross-sectional areas of flax FRP tube and plain ice or sawdust-reinforced ice core, respectively; $f_{f}$ = compressive strength of flax FRP tubes (57.2 MPa in this study) obtained by the compression tests on hollow flax FRP rings based on the actual thickness $t_{a}$ (Table 1). β is a reduction factor proposed based on the consideration that the hollow FRP rings and ice cores do not simultaneously reach their axial peak loads. The compressive strength of outer FRP tube in composite columns will be overestimated if the bearing capacity of hollow FRP rings is directly used. $f_{ic}^{\prime }$ is the compressive strength of the confined plain ice and sawdust-reinforced ice, which is obtained through regression analysis (Figure 17). It was noticed from Figure 17 that the slope of trend line for PIFFT specimens was larger than that observed for SIFFT specimens, which might be attributed to the fact that the plain ice with relatively weak compressive strength is much more sensitive to the lateral confinement from flax FRP tubes compared with sawdust-reinforced ice.

(2) $$\frac{f_{ic}^{\prime }}{f_{io}^{\prime }}=\{\begin{array}{ll} 1+1.93\frac{f_{l}}{f_{io}^{\prime }} & \mathrm{for}\;\mathrm{PIFFT}\\ \;1+\frac{f_{l}}{f_{io}^{\prime }} & \mathrm{for}\;\mathrm{SIFFT} \end{array}$$

in which $f_{io}^{\prime }$ = compressive strength of the unconfined plain ice or sawdust-reinforced ice, with values of 2.77 and 4.28 MPa, respectively, in this study; $f_{l}$ = maximum confining stress of flax FRP tubes at rupture and it is given by

(3) $$f_{l}=\frac{2E_{f}\varepsilon_{h,\mathrm{rup}}t}{D}$$

in which $E_{f}$ = hoop tensile elastic modulus of flax FRP tube; t = nominal thickness of flax FRP tube; D = diameter of confined ice; $\varepsilon_{h,\mathrm{rup}}$ = hoop rupture strain of flax FRP tube and it can be determined by

(4) $$\varepsilon_{h,\mathrm{rup}}=k_{\varepsilon }\varepsilon_{f}$$

in which $\varepsilon_{f}$ = ultimate tensile strain of flax FRP laminates obtained via flat coupon tests; $k_{\varepsilon }$ = FRP efficiency factor initially proposed by Pessiki et al. [49] for FRP-confined concrete, which was used in this study to evaluate the utilization rate of the strain capacity of the flax FRP in PIFFT and SIFFT columns. Figure 18 shows that the mean values of $k_{\varepsilon }$ were 0.313 and 0.643, respectively, for PIFFT and SIFFT specimens. 

**Figure 19:**
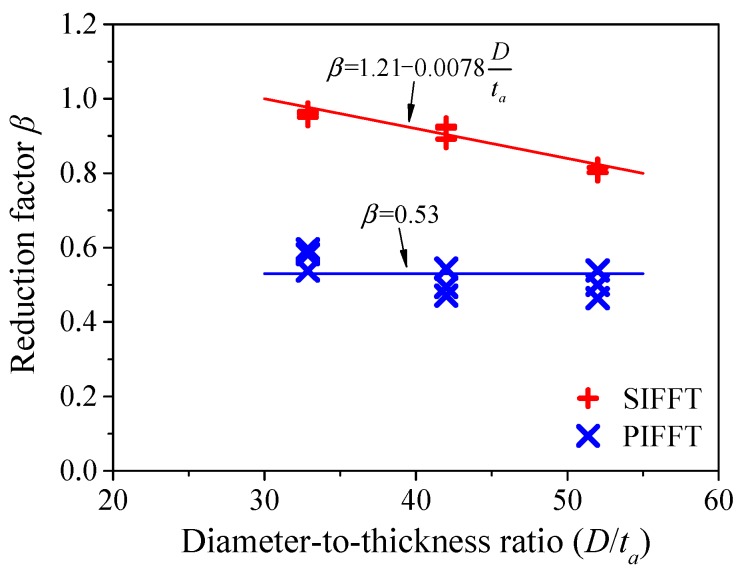
Relationship between *β* and *D*/*t_a_* in PIFFT and SIFFT specimens.

It can be seen from Figure 8 and Figure 9 that the peak axial strains of the confined plain ice and sawdust-reinforced ice in corresponding PIFFT and SIFFT short columns were smaller than those of flax FRP rings, which indicated that it was unsuitable to predict the axial bearing capacity of the composite short column using the simple superposition of the peak loads of the confined ice and FRP ring. Therefore, a reduction factor β was incorporated to assess the compressive strength of the outer flax FRP tube in PIFFT and SIFFT specimens more accurately. It was defined as the ratio of axial load of flax FRP ring corresponding to peak strain of the composite short column to the bearing capacity of flax FRP ring. Figure 19 shows the relationship between the reduction factor β and the diameter-to-thickness ($D/t_{a}$) ratio of flax FRP tube. The value of β was around 0.53 for PIFFT short columns and approximately linearly decreased with $D/t_{a}$ ratio for SIFFT short columns. The value of β was determined using Equation (5).

(5) $$\beta =\{\begin{array}{ll} 0.53 & \mathrm{for}\;\mathrm{PIFFT}\\ 1.21-0.0078\frac{D}{t_{a}} & \mathrm{for}\;\mathrm{SIFFT} \end{array}$$

**Figure 20:**
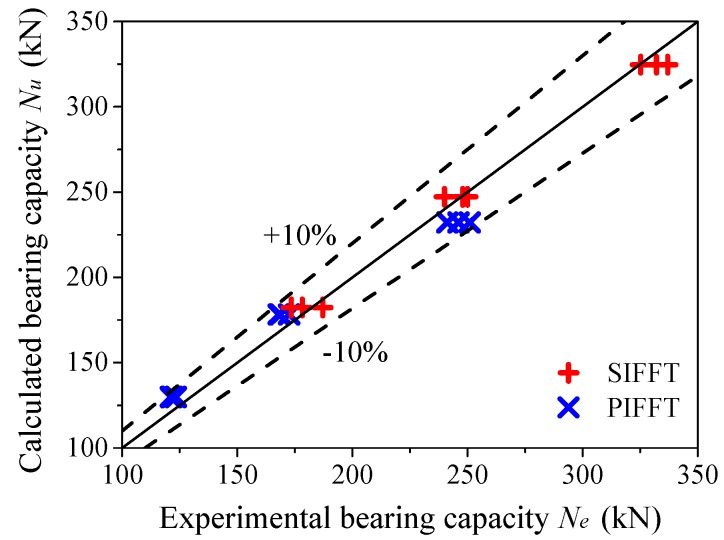
Comparison of ultimate bearing capacities between calculated and experimental results.

Figure 20 shows the comparison of ultimate bearing capacities between predicted and experimental results. The ratio of the calculated ultimate bearing capacities $N_{u}$ to the experimental results $N_{e}$ was close to one, which indicated that the proposed equation achieved good evaluation of the ultimate bearing capacities of circular PIFFT and SIFFT short columns. It is noteworthy that the equations for evaluating the bearing capacities of PIFFT and SIFFT stub columns are proposed based on the test results of fairly limited specimens tested in this study. Therefore, the accuracy of these equations remains to be verified by more test results reported by other researchers. However, to the best of the authors’ knowledge, composite columns consisting of natural FRP composites (e.g., flax FRP) and ice have not been documented in the literature. Therefore, more reasonable equations with enough accuracy need to be established on the basis of more detailed experimental results and more in-depth theoretical analysis in future study. In addition, it should be noted that axial compression tests are very sensitive to boundary conditions, which would cause non-pure compressive state in the specimens. Furthermore, the lateral confining stress provided by the flax FRP tube further complicates the internal stress in the ice core of the specimens. In order to take these factors into consideration, finite element modeling might be a good technique that could be used to study and identify this complex internal stress state of the confined ice. The related work will be incorporated in the authors’ future study.

## 5. Conclusions

This paper presents an exploratory investigation on the axial compressive behavior of circular sawdust-reinforced ice-filled flax FRP tubular (SIFFT) short columns. In addition to SIFFT short columns, three types of short columns including the plain ice specimens, the sawdust-reinforced ice specimens, and the plain ice-filled flax FRP tubular (PIFFT) specimens were also tested to make comparisons between different specimen series. It confirmed that the proposed SIFFT columns hold great potential to be used as compression members in cold areas due to its good compressive behavior. Moreover, the SIFFT column is innovatively achieved by three types of recycled constituent materials (i.e., flax fiber, ice, and sawdust), which might lead to a more sustainable and environmentally friendly solution. The results and discussions presented in this study allow the following conclusions to be drawn:

(1) Both PIFFT and SIFFT specimens fail by the rupture of flax FRP tubes outside the additional confinement zone. The lateral dilation and the development of cracks of the ice cores are effectively suppressed by the outer flax FRP tubes. Overall, the PIFFT and SIFFT specimens exhibit relatively brittle behavior.
(2) Different from the typically bilinear curves of FRP-confined concrete, the axial stress vs. strain curves of flax FRP-confined ice are quite linear. The compressive strength of confined plain ice and sawdust-reinforced ice is approximately linearly enhanced with the increasing number of FRP layers in flax FRP tubes. Sawdust-reinforced ice cores have greater compressive strength, ultimate axial strain, and energy-absorption capacity compared with plain ice cores confined by flax FRP tubes with the same thicknesses.
(3) At a given axial strain, the lateral dilation of confined sawdust-reinforced ice is smaller than that of confined plain ice. The hoop strain distribution in SIFFT specimens is more non-uniform than that observed in PIFFT specimens.
(4) The melting of plain ice specimen is effectively delayed by the inclusion of sawdust and the insulation of outer flax FRP tube, while the melting rate of unconfined sawdust-reinforced ice is close to that of confined sawdust-reinforced ice due to the insulation of a layer of dry wood fiber with a low thermal conductivity after the initial melting.
(5) Equations are proposed to estimate ultimate bearing capacities of PIFFT and SIFFT short columns with circular sections. The predictions are shown to be in reasonable agreement with the test results.

## Figures and Tables

**Table 1:** Details of specimens and main test results.

Series	Specimen	Type of Ice Core	Flax FRP Tube			Test Results			
			Ply	t (mm)	$t_{a}$ (mm)	$N_{e}$ (kN)	Average $N_{e}$ (kN)	Average $N_{f}$ (kN)	$\frac{N_{e}}{N_{i}+N_{f}}$
PI	PI0-I	PI	-	-	-	49.79	48.98	-	-
	PI0-II					48.18			
	PI0-III					48.96			
SI	SI0-I	SI	-	-	-	74.82	75.61	-	-
	SI0-II					75.62			
	SI0-III					76.38			
IF2	IF2-I	PI	2	0.27	3.00	123.27	122.00	82.66	0.927
	IF2-II					121.13			
	IF2-III					121.61			
SF2	SF2-I	SI	2	0.27	3.00	187.09	179.61	82.66	1.364
	SF2-II					178.24			
	SF2-III					173.50			
IF4	IF4-I	PI	4	0.54	3.75	168.01	169.72	103.01	1.117
	IF4-II					172.51			
	IF4-III					168.65			
SF4	SF4-I	SI	4	0.54	3.75	250.01	245.98	103.01	1.618
	SF4-II					247.95			
	SF4-III					239.98			
IF6	IF6-I	PI	6	0.81	4.86	250.94	245.39	135.56	1.330
	IF6-II					241.21			
	IF6-III					244.03			
SF6	SF6-I	SI	6	0.81	4.86	332.02	331.36	135.56	1.796
	SF6-II					325.18			
	SF6-III					336.88			

Note: t = nominal thickness of flax FRP tube without considering the thickness of resin matrix; $t_{a}$ = actual thickness of flax FRP tube; $N_{e}$ = bearing capacity of test specimens; $N_{f}$ = bearing capacity of flax FRP rings; $N_{i}$ = bearing capacity of plain ice or sawdust-reinforced ice specimens. For the type of ice core, PI = plain ice; SI = sawdust-reinforced ice.

**Table 2:** Test results of confined plain ice and sawdust-reinforced ice.

Series	Specimen	$f_{ic}^{\prime }$ (MPa)	Average $f_{ic}^{\prime }$ (MPa)	$\frac{f_{ic}^{\prime }}{f_{io}^{\prime }}$	$\varepsilon_{iu}$ (%)	Average $\varepsilon_{iu}$ (%)	$\frac{\varepsilon_{iu}}{\varepsilon_{io}}$	$\varepsilon_{h,rup}$ (%)	Average $\varepsilon_{h,rup}$ (%)	$\frac{\varepsilon_{h,rup}}{\varepsilon_{f}}$
IF2	IF2-I	4.46	4.56	1.65	1.67	1.55	1.37	0.44	0.42	0.310
	IF2-II	4.52			1.55			0.38		
	IF2-III	4.71			1.44			0.42		
SF2	SF2-I	6.78	6.37	1.49	2.65	2.63	1.09	0.91	0.84	0.628
	SF2-II	6.34			2.60			0.84		
	SF2-III	6.01			2.65			0.78		
IF4	IF4-I	6.63	6.68	2.41	1.52	1.55	1.37	0.45	0.42	0.313
	IF4-II	6.60			1.67			0.43		
	IF4-III	6.80			1.45			0.38		
SF4	SF4-I	8.78	8.60	2.01	3.02	2.99	1.24	0.81	0.85	0.633
	SF4-II	8.64			3.04			0.94		
	SF4-III	8.39			2.91			0.79		
IF6	IF6-I	9.74	9.50	3.43	1.82	1.79	1.58	0.43	0.42	0.316
	IF6-II	9.53			1.67			0.39		
	IF6-III	9.24			1.87			0.46		
SF6	SF6-I	11.43	11.40	2.66	3.35	3.35	1.39	0.90	0.90	0.668
	SF6-II	10.99			3.41			0.82		
	SF6-III	11.78			3.29			0.96		

Note: $f_{io}^{\prime }$ = compressive strength of unconfined ice (2.77 MPa for plain ice and 4.28 MPa for sawdust-reinforced ice); and $\varepsilon_{io}$ = peak axial strain of unconfined ice (1.13% for plain ice and 2.41% for sawdust-reinforced ice).
